# Pioneering the Future of Oral Healthcare: Bioprinting and Its Transformative Clinical Potential in Dentistry

**DOI:** 10.7759/cureus.79030

**Published:** 2025-02-15

**Authors:** Sumeet Agarwal, Laresh N Mistry, Shamika Kamath, Rohit Thorat, Bharat Gupta, Saba Kondkari

**Affiliations:** 1 Prosthodontics, Bharati Vidyapeeth (Deemed to be University) Dental College and Hospital, Navi Mumbai, IND; 2 Pediatric and Preventive Dentistry, Bharati Vidyapeeth (Deemed to be University) Dental College and Hospital, Navi Mumbai, IND; 3 Dentistry, NAMO Medical Education and Research Institute (MERI), Silvassa, IND; 4 Prosthodontics and Crown &amp; Bridge and Implantology, Bharati Vidyapeeth (Deemed to be University) Dental College and Hospital, Pune, IND; 5 Periodontology, MGM Dental College and Hospital, Navi Mumbai, IND; 6 Dentistry, Bharati Vidyapeeth (Deemed to be University) Dental College and Hospital, Navi Mumbai, IND

**Keywords:** bioinks, bioprinting, innovative dentistry, regeneration, tissue engineering

## Abstract

Bioprinting is revolutionizing the field of dentistry by enabling the fabrication of complex dental tissues using advanced techniques like inkjet, extrusion-based, and laser-assisted bioprinting. These methods allow for the precise placement of cells and materials to regenerate dental pulp, periodontal tissues, alveolar bone, and temporomandibular joint structures. Hydrogels, composite bioinks, and cell-laden bioinks play a crucial role in scaffold formation and improving cell viability. Preclinical models have demonstrated the potential of bioprinting for tissue regeneration and dental implants, with early clinical trials showing promising results. However, challenges remain, including scalability, material selection, immune response, and regulatory approval. Future advancements in multi-material bioprinting, real-time monitoring, and personalized treatment approaches will expand clinical applications of bioprinting, driving innovation in oral healthcare and improving patient outcomes.

## Introduction and background

Bioprinting, also known as 3D bioprinting, combines 3D printing with biomaterials to create structures that mimic natural blood vessels, bones, and tissues. Initially used in pharmacological research, it has expanded to include cell scaffolds for regenerating injured joints and ligaments. Since 2007, bioprinting has been applied in medicine to study and replicate various tissues, cartilage, and organs. In dentistry, bioprinting has revolutionized tissue engineering by enabling the layer-by-layer fabrication of biologically relevant tissues using bioinks (composed of living cells, growth factors, and biomaterials) [1]. This technology holds promise for regenerating dental tissues, designing personalized dental implants, and creating complex tissue structures that closely replicate the natural tissues in the oral cavity [2]. Research focuses on identifying trends, critically evaluating challenges, and exploring the transformative potential of bioprinting in revolutionizing dental procedures and patient care [1,2]. 3D printing, also known as additive manufacturing, has significantly impacted many sectors, particularly dentistry, due to its capacity to provide highly customized and cost-effective solutions. With previously unheard-of levels of accuracy, personalization, and efficiency, 3D printing has completely transformed the manufacturing of several prostheses, implants, and orthodontic devices in the dental field. The efficient and personalized manufacturing of crowns, bridges, and dentures represents one of the field’s most significant advancements [3].

## Review

Materials and techniques used in bioprinting

Inkjet Bioprinting

Inkjet bioprinting is widely used for bioink droplet deposition onto a substrate to construct tissue models. Its advantages include low cost and high printing speed. However, one drawback of this technique is that high cell densities can cause nozzle clogging [1,2]. Although inkjet printing was initially introduced by paper printing companies in the early 1950s, it was not until 2003 that the first patent for a printer that was remodeled to bioprint living cells became available [1]. Xia et al. suggest that using solutions with lower cell concentrations may be a constraint when assembling materials into 3D structures [4]. Xu et al. report that high spatial precision, ranging from 50 to 300 μm, can be achieved [5]. However, print resolution can be compromised by cell aggregation within the bioink, which affects droplet formation and velocity [5].

Extrusion-Based Bioprinting

Extrusion-based bioprinting is one of the most commonly used methods, where bioink is continuously extruded through a nozzle using a screw, piston, or pneumatic system to form 3D structures. The wide variety of biomaterials used for printing, such as cell-laden hydrogels, natural and synthetic polymers, and cell aggregates, makes it a preferred approach for creating multilayer scaffolds in tissue engineering [6]. The improvement of cellular organization and the mechanical properties of constructs has been facilitated by modulating the deposition rate and pattern of bioinks [7]. According to a study by Pati et al., cell death can occur during and after printing due to the pressure drop during extrusion via a micro-nozzle [8]. This issue can be mitigated by optimizing factors such as material concentration, nozzle pressure, and diameter. However, compared to inkjet and laser-based bioprinting, extrusion-based printing has a lower resolution, typically around 200 μm [8].

Laser-Assisted Bioprinting 

Laser-assisted bioprinting utilizes a focused laser beam to deposit bioink onto a substrate to allow high-resolution printing of complex tissue geometries. The effects of the laser on the cells remain unknown, despite the fact that this method is known to maintain high cell viability during printing [9]. Factors such as alginate gelation and concentration, gelation time, and laser fluence have been considered in relation to cell survival. After 24 hours of incubation, it was found that prolonged gelation periods reduced cell viability, likely due to the thick gel walls limiting nutrient and oxygen diffusion [9]. Research studies have shown that laser-assisted bioprinting can produce dental constructs with high cell viability and functionality. It has previously been demonstrated that certain cell lines are resistant to damage during the laser-assisted printing process [10]. Additionally, structures resembling tooth or periodontal tissues can be successfully bio-fabricated in preclinical models using this technique, which holds great promise for future applications [11].

Bio inks for dental applications

Hydrogels

Hydrogels are a family of cross-linked polymeric materials capable of absorbing and retaining significant amounts of water. In tissue engineering, hydrogels are classified into two groups: synthetically created hydrogels, such as polyethylene glycol (PEG) or pluronic, and naturally derived hydrogels, such as gelatin, fibrin, collagen, chitosan, and alginate [12]. In particular, naturally derived hydrogels, particularly chitosan, alginate, hyaluronan, collagen, and agarose, are highly attractive due to their inherent biocompatibility, biodegradability, and safety. Globally, these hydrogels are commonly sourced from renewable resources such as microbes, plants, animals, and algae. Synthetic hydrogels, on the other hand, offer customizable properties that facilitate the creation of practical applications [13]. Recent advancements in hydrogel bioinks focus not only on their mechanical properties but also on the integration of bioactive molecules to enhance tissue regeneration [14]. Natural hydrogels offer a more appropriate and favorable environment for cell adhesion, expansion, division, and renewal due to their superior biological compatibility, protection, and biodegradability. However, their widespread use is severely limited by issues such as low solubility, costly manufacturing processes, and regulatory challenges related to their physical and chemical properties. In contrast, synthetic hydrogels offer the advantage of tunable chemical properties, though residual monomers or degradation products can be harmful, limiting their interaction with cells and tissues [15].

Composite Bioinks

Composite bioinks are blends or multiphase materials composed of two or more polymers, encapsulated cells, and bioactive inorganic fillers [16]. These bioinks show great potential for creating constructs with properties closely resembling those of natural dental tissues (including vascular, cartilage, adipose, and bone tissues) [17].

Cell-Laden Bioinks

3D bioprinting technologies are an emerging technique in tissue engineering, allowing for the incorporation of living cells into bioactive materials to create 3D in vitro models that replicate the extracellular matrix (ECM) and advance our knowledge of physiological systems [18]. Cell-laden bioinks, which contain living cells dispersed within a biocompatible matrix, are particularly useful for the direct printing of tissue constructs with cellular components. Previous efforts have focused on optimizing the formulation of cell-laden bioinks to improve cell viability and functionality [19].

Applications of bioprinting in dentistry

Dental Pulp Regeneration

Dental pulp is essential for the vitality and health of a tooth. Recent studies have shown that it is possible to successfully bioprint dental pulp constructs using cell-laden bioinks containing dental pulp stem cells (DPSCs) and growth factors, such as vascular endothelial growth factor (VEGF). These constructs have demonstrated promising results in forming vascularized pulp-like tissues in the preclinical models, complete with functional odontoblasts [20]. A study by Zhou et al. indicated that, in order to restore dentin and form neovascular-like structures, a dental pulp guidance construct (DPGC) containing the instructional niche was bioprinted to resemble natural teeth [20]. In order to increase the stemness features of the encapsulated DPSCs and promote predominant nucleus localization of the Yes-associated protein (YAP), GelMA-Dextran aqueous emulsion was utilized as an ink for the in situ printing of porous DPGC. Additionally, the microporous DPGC-encapsulated DPSCs showed improved migration, spreading, and viability. Additionally, the study found that DPGC could stimulate neurogenesis and encourage the development of capillary tubes. The DPGC in a mouse subcutaneous implant model was made up of porous structures that resembled dental pulp, such as odontoblasts and newly created vascular systems [20]. The study also investigated how the alginate/gelatin hydrogel scaffold extract used in 3D bioprinting affected the growth and differentiation of human dental pulp stem cells (hDPSCs). By contrasting conventional and 3D-printed Alg-Gel scaffolds, the researchers examined the impact of alginate/gelatin hydrogel scaffolds on hDPSCs. The results showed that on the 3D-printed scaffolds, hDPSCs showed improved growth and adherence. Furthermore, it was demonstrated that aqueous extracts from these 3D-printed scaffolds more successfully stimulated osteogenic/odontoblastic differentiation and cell proliferation, as evidenced by the increased bone-like nodule formation, increased alkaline phosphatase activity, and upregulation of genes linked to mineralization. A more conducive milieu for hDPSC development and differentiation was shown by elemental analysis, which also showed that the 3D-printed scaffold extracts had greater quantities of calcium and phosphorus ions compared to the conventional Alg-Gel scaffold [21].

Periodontal Tissue Regeneration

Periodontal diseases are a significant cause of tooth loss and are defined by the destruction of periodontal tissues. Thus, these constructs have, so far, shown better integration with host tissues and the potential to facilitate periodontal regeneration in preclinical models [22]. Currently, clinical trials are underway to evaluate the safety and efficacy of bioprinted constructs in patients suffering from periodontal defects. Preliminary results indicate promising outcomes in tissue integration and regeneration [23]. For example, Reis et al. developed a PLGA/CaP bilayer scaffold that was implanted in a canine model of a furcation defect [24]. It had a flat-smooth exterior layer and a rough, macroporous inner layer that was 1-mm thick. The findings demonstrated the development of new periodontal ligament, bone, and cementum with Sharpey fiber insertions [24]. An intriguing overview of periodontal complex regeneration was written by Park et al. [25]. The cementum, PDL, gingiva, and alveolar bone are all components of the periodontal complex, which supports the teeth. They demonstrated a number of cutting-edge methods for periodontal regeneration, including scaffolding, cell sheet technology, and 3D printing [25].

Alveolar Bone Regeneration

Recent research has shown the feasibility of bio-fabricating alveolar bone constructs from bioinks containing osteogenic cells and growth factors like VEGF. The third molar is the best source of dental stem cells (DPSCs), which can proliferate and differentiate into osteoblast and odontoblast lineages to form dentin and bone [26]. In dentistry, dental stem cells are frequently utilized as cell sources for bone regeneration. DPSCs have been shown to have a higher osteogenic ability than bone marrow stem cells (BMSCs) and to produce vessel-integrated bone tissue structures, which are essential for the repair of large bone lesions [27]. The presented constructs were able to induce new bone formation and enhance the stability of implants in a wide range of preclinical models [2,28]. A recent clinical trial is being conducted to evaluate the use of bioprinted constructs such as alveolar bone in the replacement of knee joints, tibia, femur, fibula, and dental implant cases, with preliminary data demonstrating accelerated bone regeneration and successful integration of the implants [29].

Current research focuses on refining models to better replicate the complexity of natural dental tissues, enhancing their predictive value [30].

Scalability and standardization challenges and future directions

Current technical challenges, like scalability, multi-axis movement, and multiple printing modalities, are being addressed, and improvised versions should be available soon. Maintaining the cells' integrity throughout the drawn-out printing process will be a difficulty in the field of scaling (producing human-scale tissues and organs) [31]. Prior to becoming widely used, tissue bioprinting must overcome a number of obstacles, such as sophisticated tissue engineering, post-print maturation, scalable production, and regulatory licensing. Replicating functioning components such as lymphatics, neurological systems, and vasculature is a significant challenge. Although there is ongoing research on using microstructures to promote vascularization, problems with cell distribution and channel design still exist. Standardized libraries and quality control are necessary for repeatability and safety in the creation of bioinks. The development of bioprinting methods is also essential for scalability. Lastly, developing precise regulatory frameworks, using the FDA's 3D-printed medical device rules as a guide, is essential to clinical translation [32].

Future of bioprinting

Owing to its capacity to produce intricate organs and tissues in large quantities with accuracy and precision, this technology has been swiftly gaining ground on traditional tissue engineering techniques. It is anticipated that the global market share of 3D bioprinting will reach around $1.55 billion (USD) by the middle of the 2020s, with nearly 30% of that share coming from North America [33]. The future focus will be on the integration of these technologies with existing platforms of bioprinting for more complex, functional, and viable dental tissues [32,34]. Overtaking existing tissue engineering techniques, 3D bioprinting has the potential to completely transform the treatment of skin injuries. It provides potential remedies for burns and chronic wounds through its use in disease modeling, targeted medicine delivery, and skin regeneration. As research advances, this technique has the potential to revolutionize wound treatment, improving both healing outcomes and patient quality of life [35].

**Figure 1:**
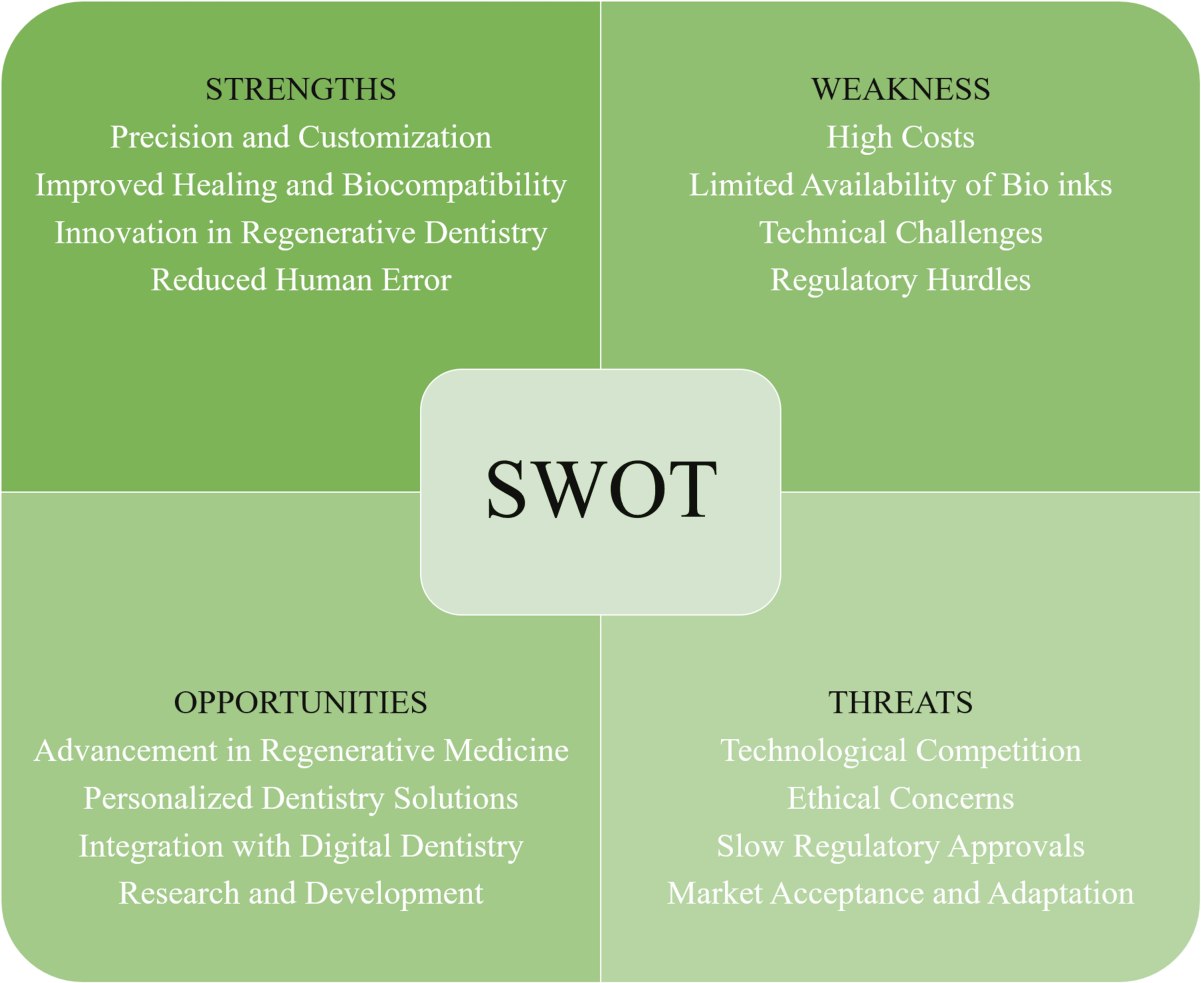
SWOT analysis SWOT: strengths, weaknesses, opportunities, and threats Image credit: Saba Kondkari

Figure 1 presents the SWOT analysis of bioprinting in dentistry.

## Conclusions

Bioprinting is at the forefront of revolutionizing dentistry through complex tissue regeneration, personalized approaches to treatment, and innovative therapeutic strategies. The recent breakthroughs in bioprinting techniques, bioinks, and clinical applications have shown great promise in addressing long-standing challenges in dental tissue engineering. Although there are numerous technical, biological, ethical, and regulatory challenges that must be overcome, ongoing research and innovation are expected to overcome these obstacles, paving the way for widespread clinical adoption. Achieving this goal will require continuous interdisciplinary collaboration to fully harness the potential of bioprinting, ultimately improving dental health and patient outcomes.
